# High-Throughput Drug Screening System Based on Human Induced Pluripotent Stem Cell-Derived Atrial Myocytes ∼ A Novel Platform to Detect Cardiac Toxicity for Atrial Arrhythmias

**DOI:** 10.3389/fphar.2021.680618

**Published:** 2021-08-03

**Authors:** Yayoi Honda, Jun Li, Aya Hino, Shinji Tsujimoto, Jong-Kook Lee

**Affiliations:** ^1^ Sumitomo-Dainippon Pharma CO., Ltd., Osaka, Japan; ^2^ Bioanalysis Group, Osaka Laboratory, Technical Solution Headquarters, Sumika Chemical Analysis Service, Ltd., Osaka, Japan; ^3^ Department of Cardiovascular Medicine, Osaka University Graduate School of Medicine, Suita, Japan; ^4^ Department of Cardiovascular Regenerative Medicine, Osaka University Graduate School of Medicine, Suita, Japan

**Keywords:** human iPS cell-derived atrial-like cardiomyocytes, nodal-like cardiomyoyctes, atrial arrhythmias, sick sinus syndrome, drug screening

## Abstract

Evaluation of proarrhythmic properties is critical for drug discovery. In particular, QT prolongation in electrocardiograms has been utilized as a surrogate marker in many evaluation systems to assess the risk of torsade de pointes and lethal ventricular arrhythmia. Recently, new evaluation systems based on human iPS cell-derived cardiomyocytes have been established. On the other hand, in clinical situations, it has been reported that the incidence of atrial arrhythmias such as atrial fibrillation has been increasing every year, with the prediction of a persistent increase in the near future. As to the increased incidence of atrial arrhythmias, in addition to the increased population of geriatric patients, a wide variety of drug treatments may be related, as an experimental method to detect drug-induced atrial arrhythmia has not been established so far. In the present study, we characterized the atrial-like cardiomyocytes derived from human induced pluripotent stem cells and examined their potential for the evaluation of drug-induced atrial arrhythmia. Atrial-like cardiomyocytes were induced by adding retinoic acid (RA) during the process of myocardial differentiation, and their characteristics were compared to those of RA-free cardiomyocytes. Using gene expression and membrane potential analysis, it was confirmed that the cells with or without RA treatment have atrial or ventricular like cardiomyocytes, respectively. Using the ultra-rapid activating delayed rectifier potassium current (I_Kur_) channel inhibitor, which is specific to atrial cardiomyocytes, Pulse width duration (PWD) 30cF prolongation was confirmed only in atrial-like cardiomyocytes. In addition, ventricular like cardiomyocytes exhibited an early after depolarization by treatment with rapidly activating delayed rectifier potassium current (I_Kr_) channel inhibitor, which induces ventricular arrhythmia in clinical situations. Here, we have established a high-throughput drug evaluation system using human iPS cell-derived atrial-like cardiomyocytes. Based on the obtained data, the system might be a valuable platform to detect potential risks for drug-induced atrial arrhythmias.

## Introduction

It has been reported that 22% of candidate compounds dropped out at the developmental stage of new drugs and 45% of pharmaceutical products were withdrawn from the market (Watkins, 2011). Therefore, in research and development, it is critical to detect cardiotoxicity during the selection of appropriate compounds for new drugs. In particular, drug-induced torsade de pointes (TdP) has been the most serious concern as it leads to ventricular fibrillation or sudden death.

In western countries, cases of TdP-related death were frequently reported in the late 80s through the early 90s. Several pharmaceutical products were withdrawn from the market because of their risk of inducing TdP in the early 90s (Thomsen et al., 2006; Stockbridge et al., 2013). The International Council for Harmonisation of Technical Requirements for Pharmaceuticals for Human Use (ICH) guidelines for cardiotoxicity evaluation (non-clinical: S7B, clinical: E14), therefore, were enforced to handle these situations, and there have been few examples of product withdrawal due to TdP after the launch of ICH guidelines (Stockbridge et al., 2013). In recent years, human induced pluripotent stem cell-derived cardiomyocytes (hiPSC-CMs) in which ventricular cardiomyocytes are dominant have been commercially available, and multi-facility validation studies using these cells to improve the predictability of drug-induced arrhythmia evaluation have been in progress (Nozaki et al., 2017; Ando et al., 2017).

Cases of not only ventricular but also atrial arrhythmia are also known in clinical situations. Atrial arrhythmia indicates some phenotypes including tachyarrhythmia such as atrial flutter or fibrillation, or bradyarrhythmia such as sick sinus syndrome or atrioventricular block. In tachyarrhythmia, atrial fibrillation occasionally induces cardiac arrest caused by a deterioration of contraction, atrial thrombus formation, and related infarction in organs/tissues. Bradyarrhythmia also causes transient cardiac arrest accompanied by loss of consciousness or cardiac arrest. Although the fatality rate caused by atrial arrhythmia itself is not very high, some lethal diseases originating from atrial arrhythmia are known. The incidence of atrial arrhythmia has increased annually in recent decades (Jensen et al., 2012; Chugh et al., 2014; Schnabel et al., 2015; Lane et al., 2017) and it is predicted that the number of patients will be growing (Jensen et al., 2014; Lane et al., 2017). Aging can be thought of as one of its risk factors, and an aging society might be related to an increase in the number of atrial arrhythmias. Several medicines, such as calcium antagonists, beta-adrenergic blockers, or antipsychotic agents, induce bradyarrhythmia (Edoute et al., 2000; Berry and Hasin, 1978; Johnson et al., 1997; Bordier et al., 2003). Not only aging but also drug treatment may be a trigger for tachyarrhythmia. As the evaluation tools for atria arrhythmia have not been established thus far, we could not confirm the existence of drug-induced atrial arrhythmia itself.

As ventricular cardiomyocytes are dominant in commercially available hiPSC-CMs, atrial arrhythmia might not be detected using these cells. Retinoic acid (RA) is essential in the regulation of cardiac development (Moss et al., 1998). Inhibition of RA synthesis induced the lack of atrial chamber in mouse embryo heart (Xavier-Neto et al., 1999). Hidaka et al.(2003) established a differentiation protocol from mouse embryonic stem (ES) cells to cardiomyocytes, and confirmed that retinoic acid induced atrial cardiomyocytes from ES cells. In recent years, retinoic acid has also been used for inducing atrial cardiomyocyte from human ES/iPS cells Zhang et al. (2011), Gassanov et al. (2008), Lee et al. (2017), and these differentiation methods have been proposed for the application of pathophysiological models of atrial arrhythmia or drug screening for atrial arrhythmia (Devalla et al., 2015; Argenziano et al., 2018; Lemme et al., 2018). In the present study, we obtained a hiPS-derived atrial-like phenotype by supplemented RA during the cardiac mesoderm induction. Accordingly, we tried to examine the acute response to drug administration in the hiPS-CMs with (atrial-like subtype) or without (ventricular-like subtype) treatment of RA.

## Results

### Cardiac Differentiation Induction From hiPSCs

**FIGURE 1:**
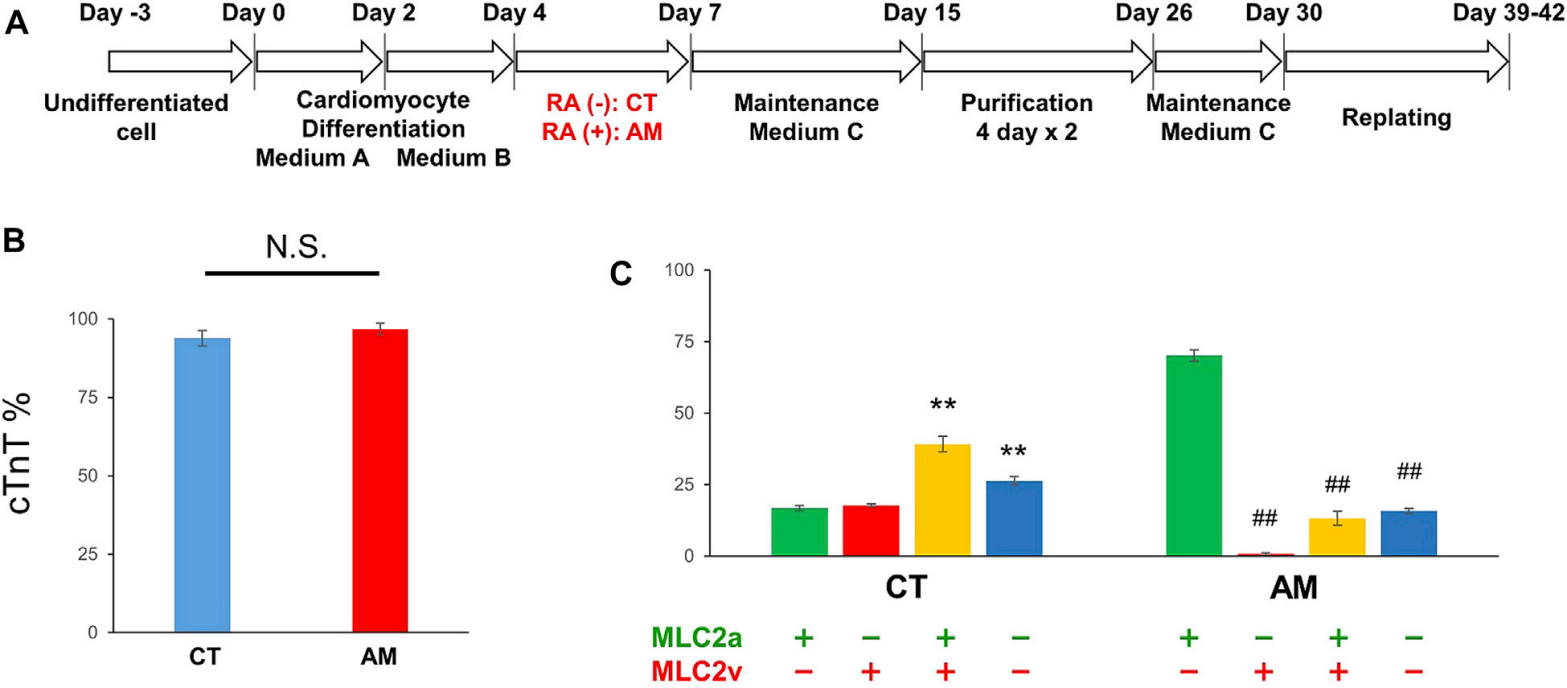
Cardiac differentiation from hiPSCs **(A)** Schematic of the protocol for the differentiation of cardiomyocytes from hiPS cells with (RA (+)) or without RA (RA (−)) treatment **(B)** The ratio of cardiomyocytes quantified by immunostaining with cardiac troponin-T (cTnT). The proportion of cTnT positive cells was 93.91 ± 2.51% in CT (control hiPS-CMs), or 96.79 ± 1.81% in AM (RA-treated hiPS-CMs), respectively (P = N.S.). Error bars represent SD of the mean from the values of independent experiments. *n* = 3. N.S., not significant **(C)** Immunofluorescence analysis of the percentage of MLC2a (atrial isoform) and MLC2v (ventricular isoform) positive expression in the CT and AM. Error bars represent SD of the mean from the values of independent experiments. **p < 0.01 for comparison with MLC 2a+/MLC 2v-cardiomyocyte in CT, ##*p* < 0.01 for comparison with MLC 2a+/MLC 2v-cardiomyocyte in AM, *n* = 3.

Undifferentiated hiPSCs were maintained in AK02 medium for 2–3 days until 80% confluency was obtained. Cardiac differentiation was then induced according to the manufacturer’s protocol, as shown in Figure 1A. To obtain highly purified cardiomyocytes, metabolic selection was performed during post-differentiation, from day 15–26. Cardiomyocyte proportion was quantified by immunostaining with cardiac troponin-T (cTnT). Figure 1B shows that the proportion of cTnT-positive cells was over 90% with or without RA treatment (CT 93.9 ± 2.51% vs. AM 96.8 ± 1.81%). The results suggest that RA application did not influence cardiomyocyte differentiation efficiency.

### Characteristics of hiPS-Derived Cardiomyocytes With RA Treatment

To further evaluate the gene expression of cardiac subtypes in the control hiPS-CMs (CT) and the RA-treated hiPS-CMs (AM), the cells were co-stained with myosin light chain 2a (MLC-2a: atrial isoform) and myosin light chain 2v (MLC-2v: ventricular isoform) (Supplementary Figure 1). An immunofluorescence study showed that the ratio of MLC-2a positive cells and MLC-2v positive cells are comparable in CT (MLC-2a 56.0 ± 2.1% vs. MLC-2v 57.0 ± 2.11%), and the percentage of double-positive cells (MLC-2a^+^/MLC-2v^+^) was approximately 40%. In AM (MLC-2a 83.4 ± 0.63%), most cTnT-positive cells were co-positive for MLC-2a, however, very few cTnT-positive cells were found co-positive for MLC-2v (MLC-2v 14.0 ± 2.82%) (Figures 1B, C).

To analyze the differences in the functional and electrophysiological properties of atrial and ventricular cardiomyocytes, we assessed multiple parameters of the action potential in CT and AM using a voltage-sensitive dye.

**FIGURE 2:**
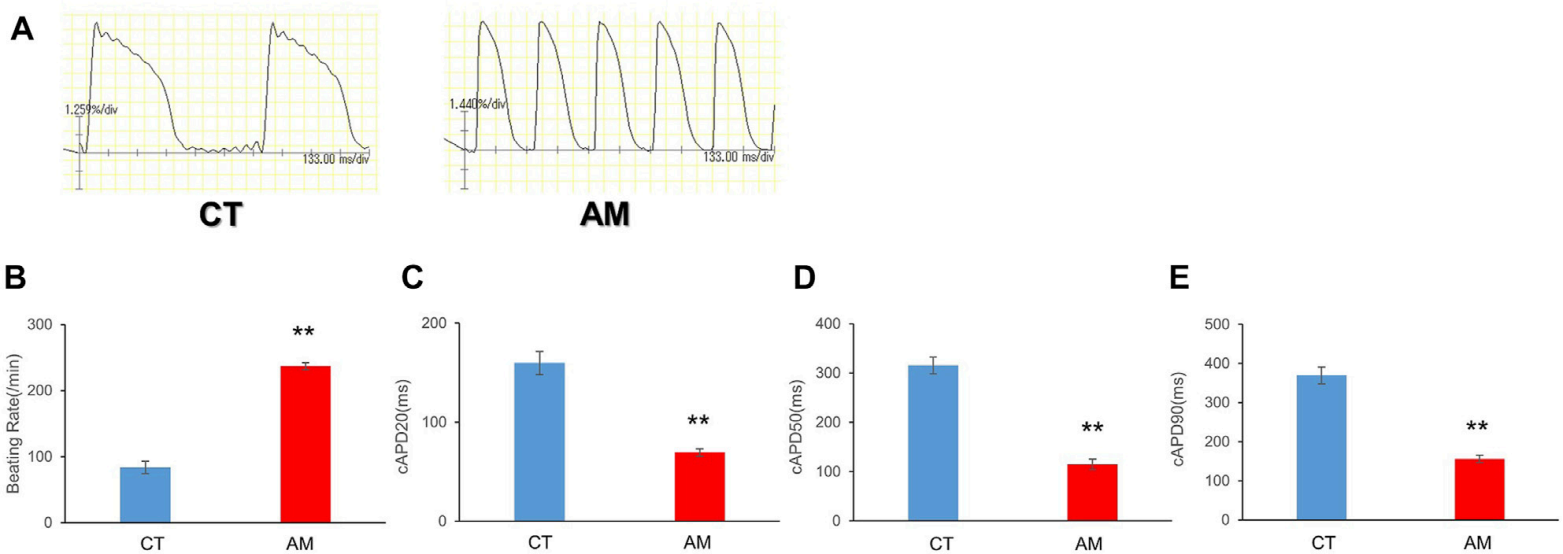
Optical action potential (OAP) of CT (control hiPS-CMs) or AM (RA treatment hiPS-CMs). Representative waveforms for AP of CT or AM. The comparison of AP parameters: beating rate, cAPD20, cAPD50, and cAPD90, between CT and AM. Error bars represent SD of the mean from the values of independent experiments. **p < 0.01, *n* = 3.

To examine the electrophysiological properties, we conducted optical mapping of action potentials based on spontaneous beating cardiomyocytes. The AP of AMs displayed a spike-like shape with a steep upslope and downslope, as well as a high frequency, which was more than twice that of CT (Figures 2A, B. CT 83.7 ± 9.50/min vs. AM 237 ± 5.20/min). In addition, the action potential duration in AM was significantly shorter than that in CT (Figures 2C–E. CT vs. AM: cAPD_20_: 159.8 ± 11.7 vs. 69.8 ± 3.66 ms; cAPD_50_ 315.5 ± 16.93 vs. 114.9 ± 10.7 ms; cAPD_90_ 369.7 ± 21.7 vs. 156.1 ± 9.19 ms).

The above data suggested more atrial-like properties in ATRA-treated AM compared with that of control CT.

### High-Throughput Drug Testing Using hiPS-Derived Atrial Myocytes

Using a high-throughput screening system (FDSS/μCell), we assessed the drug toxicity of several experimental drugs on hiPS-derived atrial myocytes as well as control hiPS-derived cardiomyocytes. Membrane potential was used as one of the parameters during the assessment.

After purification, hiPS-derived cardiomyocytes were plated in 96-well plates for downstream drug response assessment using FluoVolt™ Membrane Potential Kit. The results of the analysis of membrane potentials are summarized in Table 1. For the proper evaluate the variance of electrophysiological response in CT and AM after drug administration, we have recorded the baseline data of CT and AM respectively (Supplementary Table 1). The comparison of variance in the response of drug administration in CT and AM was represented by the percentage of changes based on their baseline values respectively.

**TABLE 1:** Effects of test compounds in MP of CT or AM. The change scopes of MP parameters are represented by the width of % change.

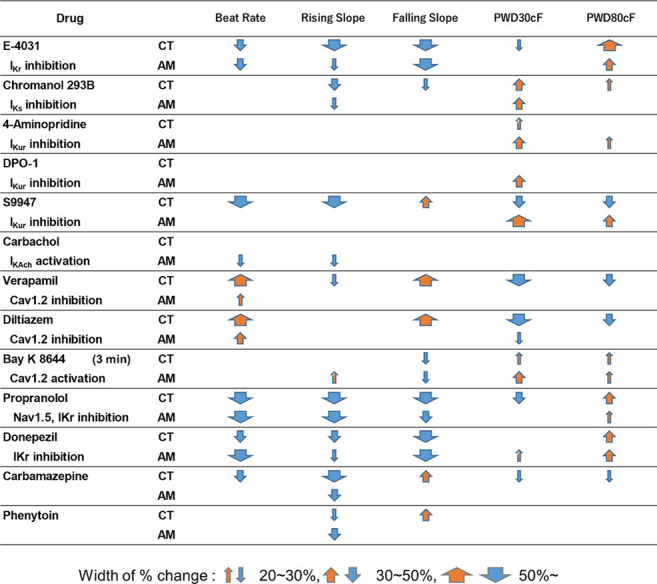

**FIGURE 3:**
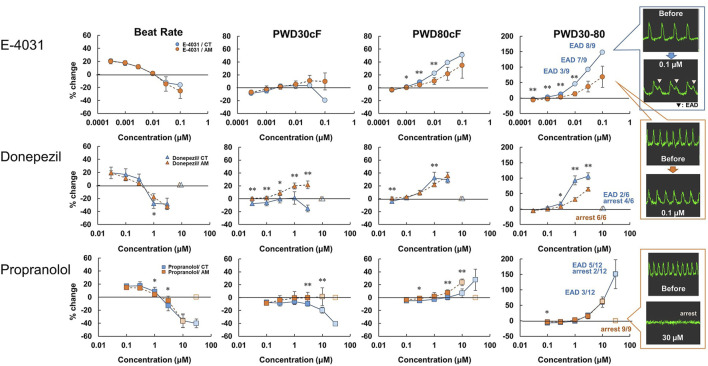
Dose-response of I_Kr_ blockers in CT and AM. CT and AM were treated with various concentrations of E-4031, donepezil, and propranolol, and membrane potential (MP) was analyzed by FDSS/μCell imaging platform. *Y*-axis represents the percentage change from the value before test compound addition. Comparison of MP parameters: beat rate, PWD30cF, PWD80cF, PWD30-80 in CT and AM. Error bars represent SD of the mean from the values of independent experiments. n = 3–12. *p < 0.05, **p < 0.01 for comparison of drug treatment of CT vs. AM (right-most panel) representative waveforms for MP in CT or AM after application of 0.1 μM E-4031. Although both CT and AM showed an increase in membrane potential duration, EAD was elicited in CT. E-4031 data: reuse from the conference paper with permission (Honda, 2020).

hiPS-derived cardiomyocytes without RA (CT) or with RA (AM) were applied with an incremental concentration of I_Kr_ blockers: E-4031, donepezil, and propranolol. The application of E-4031 (0.0003–0.1 μM), donepezil (0.03–10 μM), and propranolol (0.1–100 μM) showed a dose-dependent decrease in beating rate, rising slope, and falling slope of membrane potential in both the CT and the AM (Figure 3, Supplementary Figure 2). PWD (Pulse width duration) 80cF and PWD30-80 were prolonged as the drug concentration increased. Although under the 0.1 μM E-4031 conditions, both CT and AM showed an increase in membrane potential duration, EAD was elicited in CT (Figure 3).

As presented in Table 2, E-4031 treatment increased the incidence of EAD in a dose-dependent manner. When treated with 10 μM donepezil on CT, EAD was observed in 2 of 6 cases, while cessation of beating was observed in the remaining 4 cases. However, all of the wells appeared to stop beating in AM under the same dose of donepezil. For the application of propranolol, the CT showed irregular beating or the EAD appeared once the concentration was above 10 μM. Cessation of beating was observed in all cases at a 100 μM dose of propranolol. In the AM, 1 out of 9 samples showed irregular beating at a 10 μM dose of propranolol, and all wells ceased beating at concentrations higher than 30 μM.

**TABLE 2:** Effects of test compounds on arrhythmia-like waveforms in CT or AM. The blue frame indicates drug application concentration. Values indicate an incidence of arrhythmia-like waveforms; green column: irregular beat, yellow column: arrest, red column: EAD.

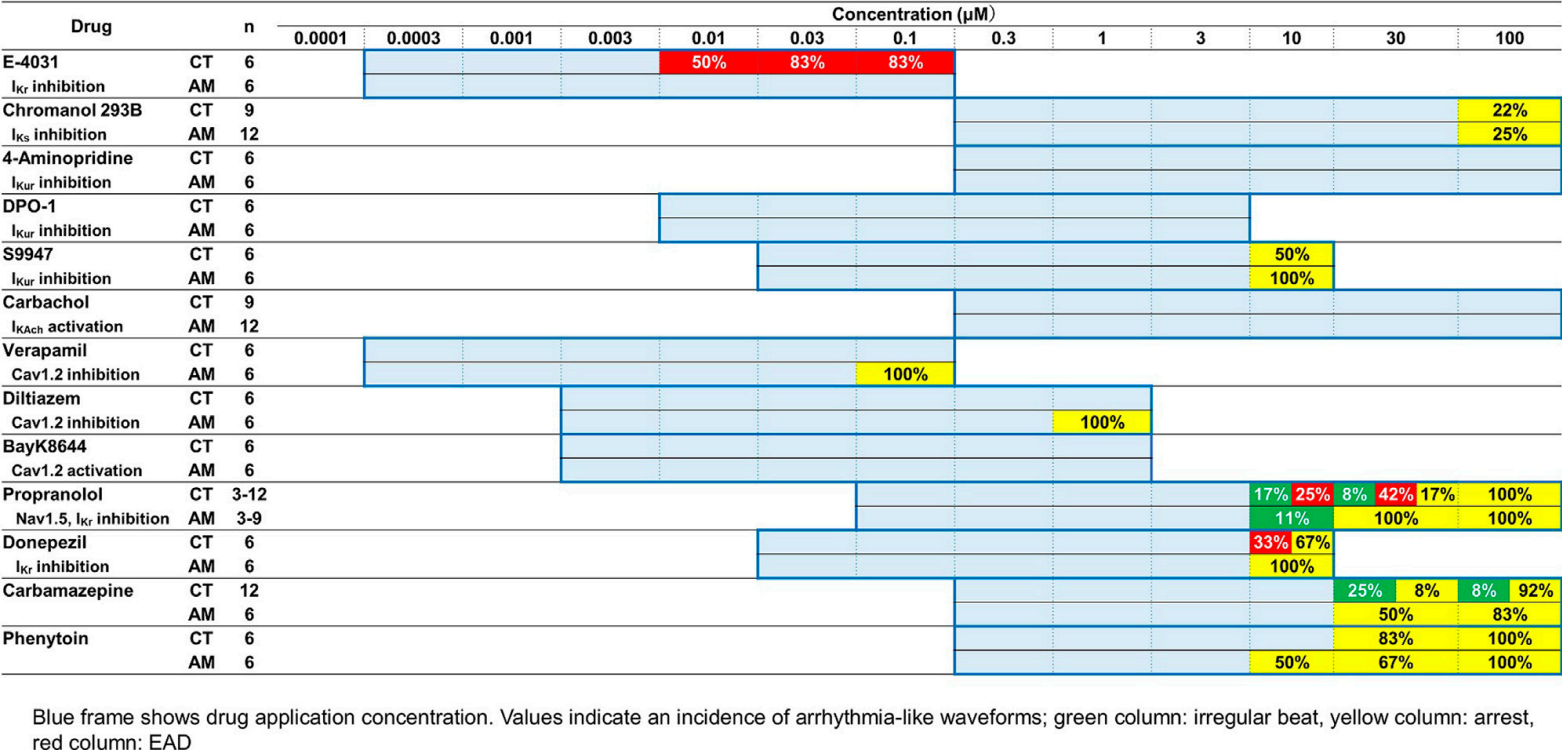

With the application of the I_Ks_ channel blocker Chromanol 293B in the range of 0.3–100 μM, CT either AM exhibited a dose-dependent decrease in the rising slope of membrane potential, while an increase in the membrane potential duration was observed (data not shown). When a dose under 100 μM of Chromanol 293B was administered, cessation of beating appeared in 2 of 9 samples in CT and 3 of 12 samples in AM.

**FIGURE 4:**
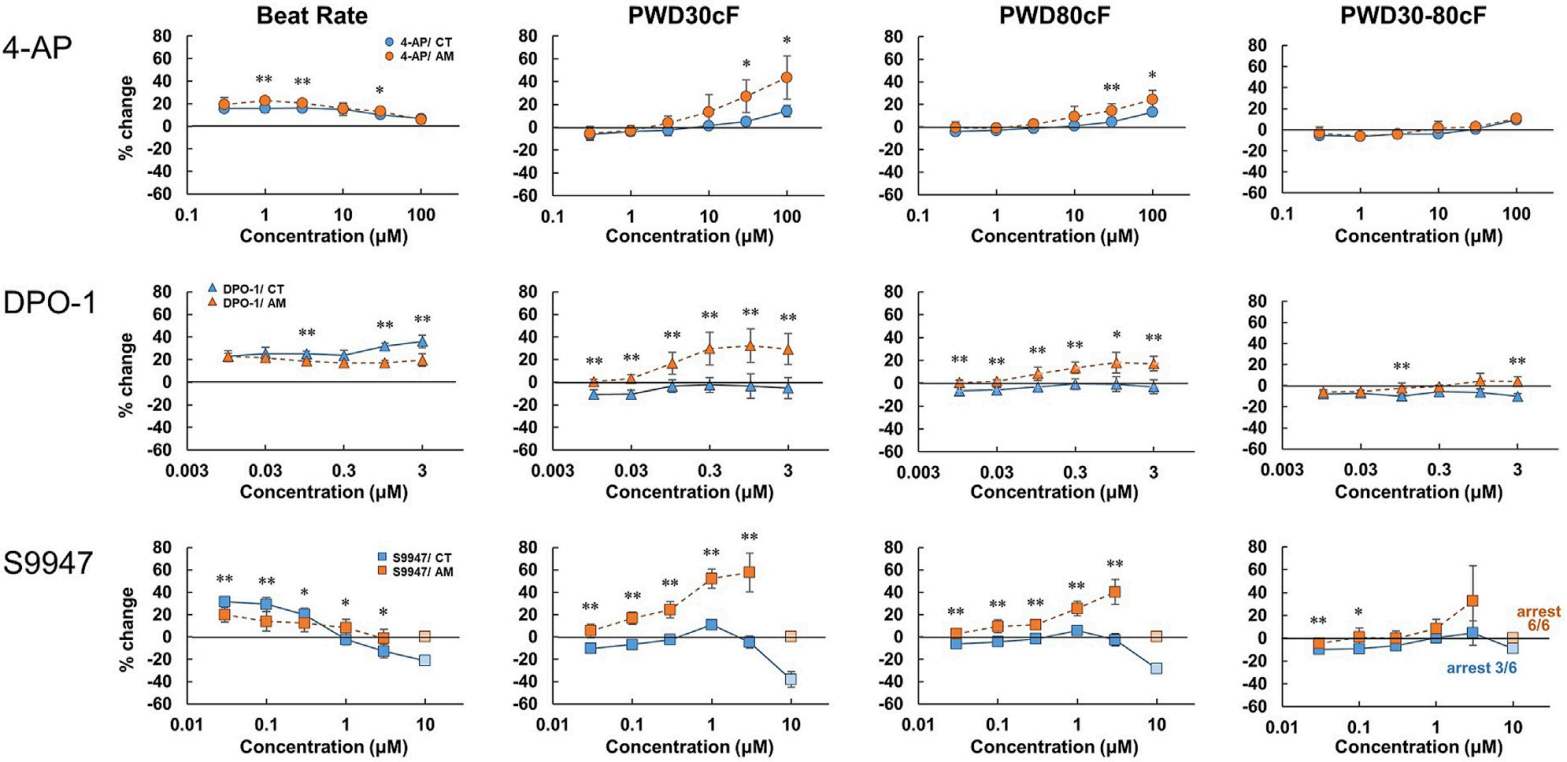
Dose-response of I_Kur_ channel blockers in CT and AM. CT and AM were treated with various concentrations of 4-Aminopiridine (4-AP), DPO-1, and S9947, and MP was analyzed by FDSS/μCell imaging platform. *Y*-axis represents the percentage change from the value before test compound addition. Comparison of MP parameters: beat rate, PWD (Pulse width duration) 30cF, PWD80cF, PWD30-80cF in CT and AM. Error bars represent SD of the mean from the values of independent experiments. n = 6, *p < 0.05, **p < 0.01 for comparison of drug treatment of CT vs. AM. DPO-1 data: reuse from the conference paper with permission (Honda, 2020).

To evaluate the response of CT or AM to the I_Kur_ channel blockers, 4-Aminopiridine (4-AP) (0.3–100 μM), DPO-1 (0.01–3 μM), and S9947 (0.03–10 μM) were used within various dose ranges, respectively (Figure 4). In AM, the application of 4-AP and DPO-1 exhibited dose-dependent increases in PWD30cF and PWD50cF. S9947 treatment of AM led to a dose-dependent increase in membrane potential duration, and was the highest in PWD30cF. All of the AM samples were arrested after application of 10 μM of S9947, although the half cases were arrested on CT accompanied by a decrease in the amplitude of membrane potential.

Carbachol, which can activate I_KAch_ current, led to a decrease in the beating rate and rising slope of membrane potential in AM; however, no significant difference was observed in the response to CT (Table 1). No irregular beating or EAD was elicited on CT either in AM.

**FIGURE 5:**
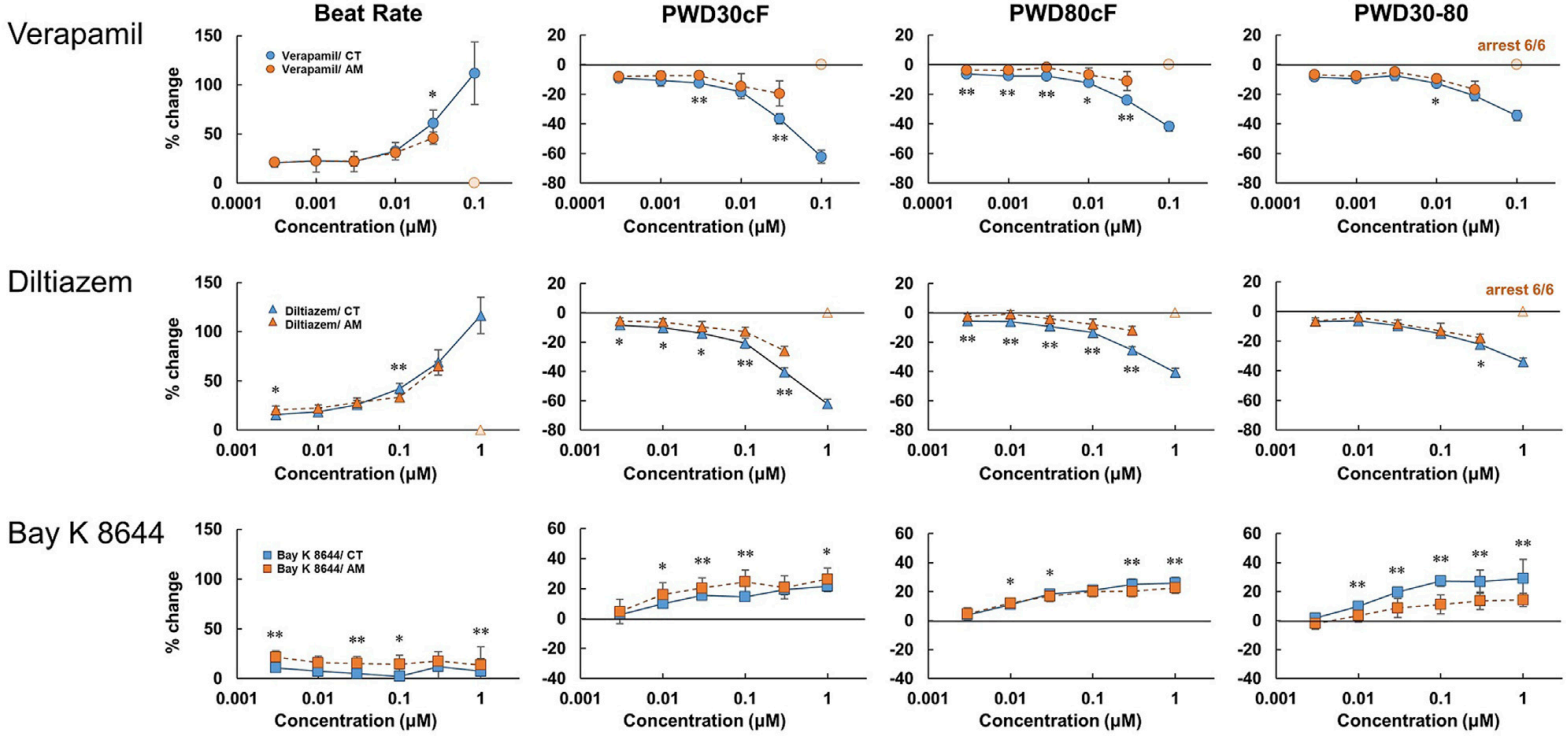
Dose-response of calcium ion channel regulator in CT and AM. Comparison of MP parameters: beat rate, PWD30cF, PWD80cF, PWD30-80 in CT and AM after application of calcium ion channel blocker verapamil and diltiazem, as well as the selective calcium channel activator Bay K 8644. Error bars represent SD of the mean from the values of independent experiments. *n* = 6. *p < 0.05, **p < 0.01 for comparison of drug treatment of CT vs. AM. Bay K8644 data: reuse from the conference paper with permission (Honda, 2020).

As widely used drugs for cardiovascular diseases, the calcium ion channel blocker verapamil and diltiazem, as well as the selective calcium channel activator Bay K8644, were also tested at various concentrations (Figure 5, Supplementary Figure 3). For CT, verapamil and diltiazem showed dose-dependent increases in beating rate and falling slope of membrane potential. Accordingly, the membrane potential durations decreased, especially for PWD30cF and PWD50cF. However, in AM, except for the dose-dependent increase in the beating rate, no obvious changes were observed in other parameters of membrane potential. At 0.1 μM doses of verapamil and diltiazem, all the AM samples showed cessation of beating. In contrast, Bay K8644 showed a dose-dependent decrease in the falling slope of membrane potential and prolonged membrane potential duration in both CT and AM samples. The CT either AM showed an acute response to Bay K8644, and PWD30 prolonged significantly (Table 1).

**FIGURE 6:**
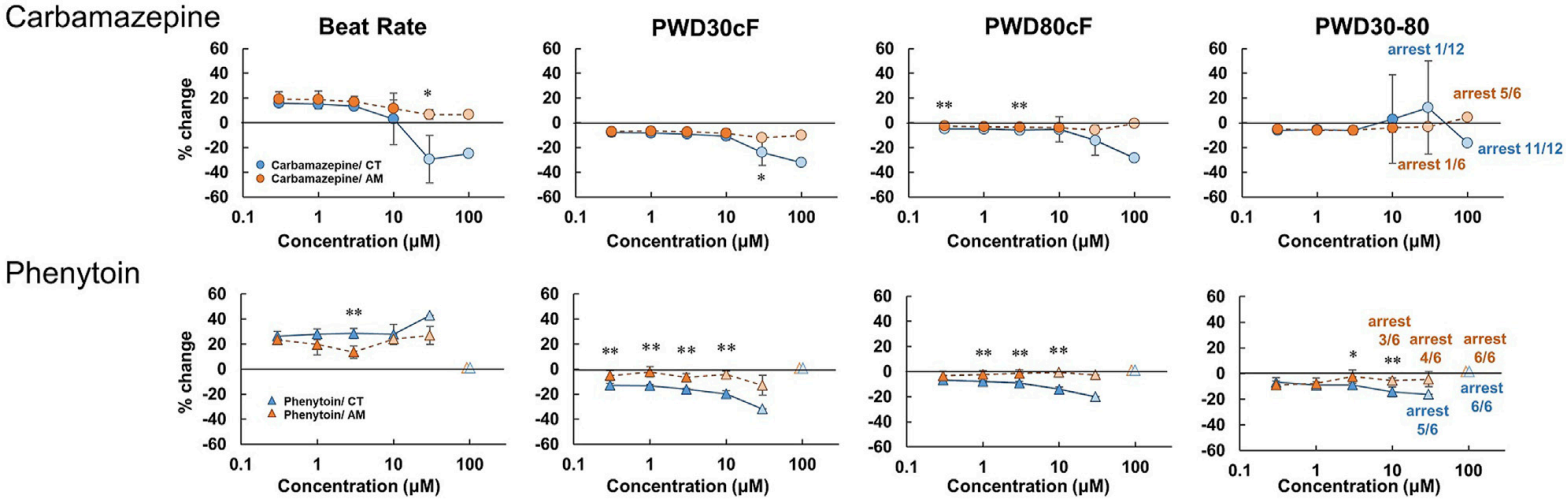
Dose-response of sodium ion channel regulator in CT and AM. Comparison of MP parameters: beat rate, PWD30cF, PWD80cF, PWD30-80 in CT and AM after application of carbamazepine and phenytoin. Error bars represent SD of the mean from the values of independent experiments. n = 6–12. *p < 0.05, **p < 0.01 for comparison of drug treatment of CT vs. AM.

Carbamazepine treatment in CT led to a decrease in the beating rate and the rising slope of membrane potential, as well as an increase in falling slope and shortened membrane potential duration in a dose-dependent manner (Figure 6, Supplementary Figure 4). Irregular beating and cessation of beating were induced over application of concentrations above 30 μM. At 100 μM, 11 of 12 cases in CT showed arrest of beating (Table 2). In contrast, in AM, no obvious change was observed except a decrease in the rising slope of the membrane potential. However, cessation of beating was observed in half the cases and 5 of 6 cases at concentrations of 30 μM and 100 μM, respectively (Table 2).

The effect of phenytoin on CT was characterized by a dose-dependent decrease in the rising slope and an increase in the falling slope of membrane potential (Figure 6, Supplementary Figure 4). Cessation of beating was elicited when the concentration was higher than 30 μM (Table 2). In AM samples, there was also a dose-dependent decrease in the rising slope of membrane potential, though cessation of beating was elicited at the concentration higher than 10 μM. Furthermore, the incidence of cessation of beating increased in a dose-dependent manner (Table 2).

## Discussion

In the present study, we established a high-throughput drug testing system using human iPS cells derived atrial-like myocytes based on our previous reports (Nakanishi et al., 2019; Hidaka et al., 2003).

The protocol successfully yielded atrial-like myocytes with adequate quality and efficiency, similar to the previous report by (Argenziano et al., 2018).

In RA-treated myocytes, gene expression levels showed a decrease in ventricle-specific transcription factor IRX4 and an increase in atrium-specific NPPA. On the other hand, those of cardiac ion channels such as Ca^2+^ and K^+^ channels, which constitute action potentials, were expressed in both groups, while atrial specific channels such as KCNA5 and KCNJ3 were significantly increased in RA-treated myocytes. These results suggest that RA treatment induces atrial differentiation, similar to previous reports (Devalla et al., 2015; Argenziano et al., 2018; Lemme et al., 2018; Nakanishi et al., 2005).

The measurement of cardiac action potentials has been used in the research of disease modeling and drug discovery based on hiPS-derived cardiomyocytes, since APs directly recapitulate patients’ pathophysiological conditions and reflect the efficacy and cardiotoxicity of pharmacological compounds as drug candidates. Although patch-clamp techniques have been widely used for single-cell-based precise electrophysiological testing, the technique is not suitable for high-throughput screening systems. Thus, in the present study, we employed optical recording using membrane potential dyes (FluoVolt™).

As shown in Figure 2, the optical action potentials of CT demonstrated the “plateau” phase, while the shorter action potentials of AM demonstrated a lack of “plateau.” This property was recognized as the difference of the ratio APD_30–40_/APD_70–80_ in CT (1.30 ± 0.18) and in AM (0.75 ± 0.11) (data not shown). Although the data in the present study was less than the data reported by Argenziano et al. (APD_30–40_/APD_70–80_: 0.97 ± 0.12 in CT and AM 0.48 ± 0.0), this may be ascribed to the difference in beating rates (Argenziano et al., 2018).

Based on these properties, hiPS-CTs and hiPS-AMs can be considered stable platforms for electrophysiological testing for drug discovery and development, although the cells may have an immature nature compared to native cardiomyocytes, as reported previously (Feric and Radisic, 2016).

### I_Kr_ Blockers

We further verified the feasibility of detecting atrial-specific responses of various pharmacological compounds. In the present study, we examined the effect of E-4031 (an I_Kr_ blocker) on OAP parameters in CT and AM As a result, E-4031 caused dose-dependent increases in PWD80cF and PWD30-80, and a decrease in the rising slope and falling slope in both CT and AM (Figure 3, supplementary Figure 2). These results are comparable to the previous report by Knobloch stating that I_Kr_ blockers prolonged ventricular repolarization and the atrial refractory period (Knobloch et al., 2002). In addition, it is known that I_Kr_ blockers cause QT prolongation and induce ventricular arrhythmias. Thus, previous papers reported that I_Kr_ blockers showed characteristic proarrhythmic changes in action potentials in ventricular-rich-hiPS-CMs (Ma et al., 2011; Nozaki et al., 2017). In the present study, since EADs were observed in CT but not in AM (Figure 3; Table 2), induction of proarrhythmic propensity by I_Kr_ blockers in the hiPS-based platform was a ventricular specific phenomenon. Based on this observation, our platform clearly showed the difference between control (induction of arrhythmias) and atrial platform (inhibition of arrhythmias).

### Donepezil (Anti-dementia Drug)

Donepezil is a choline esterase inhibitor used to treat Alzheimer’s disease and dementia with Lewy bodies, and the drug shows I_Kr_ blocking action with various critical side effects including QT prolongation and lethal ventricular arrhythmias (Kho et al., 2021). Our present data showed that donepezil prolonged the membrane potential duration at a concentration consistent with previous reports 1.3–9.9 μM (Chae et al., 2015) (Figure 3). It has been also reported that the drug causes bradyarrhythmias (Rosenbloom et al., 2010; Kuwahata et al., 2021). In the present study, consistent with the reports, the drug induced the cessation of spontaneous beating in AM and CT at the concentration higher than 30 μM (Table 2).

### I_Kur_ Blockers

I_Kur_ channels are expressed specifically in the atrium and contribute to atrial repolarization by activating in an ultrarapid manner. I_Kur_ channels have attracted attention because the channel has been considered as a pharmacological target for atrial tachyarrhythmias such as atrial fibrillation (Courtemanche et al., 1999; Nattel et al., 2002). DPO-I, an I_Kur_ inhibitor, showed prolongation of action potentials in human atrial myocytes but not in ventricular myocytes (Lagrutta et al., 2006). In addition, a previous study reported that S9947, another I_Kur_ inhibitor, did not affect ventricular function and porcine ventricular myocytes, but it prolonged the atrial refractory period (Knobloch et al., 2002). In the present study, consistent with the previous reports, DPO-1, S9947, and 4AP (non-selective I_Kur_ inhibitor) showed prolongation in PWD30cF and PWD50cF, but not in CT (Figure 4). In addition, our results showed that the KCNA5 gene was more abundantly expressed in AMs than in CT (data not shown). These results suggest that the present system is feasible for detecting the effect of pharmacological compounds on the early repolarization phase through I_Kur_ channels.

### Bradyarrhythmias

As for cardiac toxicity, in addition to QT prolongation related to lethal ventricular arrhythmias, life-threatening bradyarrhythmias are also critical rhythm disorders to be avoided. Gene expression patterns in AM suggested that the present system not only shows atrial characteristics but also nodal type cells. Our previous paper reported that retinoic acid-treated hiPS-CMs demonstrated nodal type properties as well as atrial type properties in the gene expression of SHOX2 and HCN4 (Nakanishi et al., 2019). Thus, AM would be useful to detect unexpected electrophysiological action of pharmacological compounds. In the present study, AM demonstrated not only atrial properties but also nodal type, as demonstrated by the increase observed in SHOX2 gene expression and spontaneous beating rates. In this regard, the present system would be useful to detect drug toxicity to induce critical rhythm disorders such as sick sinus syndrome.

### I_KAch_ Agonists

I_KAch_ channels are expressed specifically in the atrium as well as I_Kur_, and contribute to atrial repolarization and resting membrane potentials. The activation of the channel causes shortening and increased dispersion of refractory periods, and these induce atrial reentry following the onset of atrial fibrillation (Liu et al., 1997). In the present study, carbachol, an I_KAch_ agonist, showed a decrease in beating rate and rising slope in AM but not in CT. In the present study, we analyzed the data at the time point (10 min after addition of the drug); however, carbachol caused irregular beats early after application only in AMs (data not shown). This phenomenon suggests that carbachol-induced activation of I_KAch_ led to the increased dispersion of the refractory period, and thus, the optimal analysis time remains to be re-evaluated. In the present system, it is now feasible to evaluate the propensity for drug-induced tachyarrhythmias by comparing the chamber-specific effect of cardiac ion channels of pharmacological compounds.

### Ca^2+^ Channel Blockers and Agonist

In the present study, we tested Ca^2+^ channel blockers (verapamil and diltiazem) and an agonist (Bay K 8644). As a result, both verapamil and diltiazem decreased membrane potential duration through the shortening of the plateau phase. On the other hand, Bay K 8644 prolonged membrane potentials, especially PWD30cF in AM as well as in CT. These responses are different from those seen in I_Kur_ blockers, which showed prolongation in AM, but not in CT. In addition, more strikingly, both verapamil and diltiazem caused arrest of spontaneous beating in all the tested samples in the AM at the maximal concentration (Table 2). The results may be ascribed to the involvement of nodal type cell type in AMs in which the Ca current plays an important role in generating pacemaker potentials.

### Carbamazepine (Anti-epileptic Drug)

Carbamazepine is an antiepileptic drug that blocks neural Na channels, but the drug is also known to block the cardiac Na^+^ channel (Nav1.5) with an IC_50_ of 152.0 μM (Harmer et al., 2011). In the present study, the drug showed a decrease in the rising slope and amplitude of membrane potentials in CT (Figure 6, supplementary Figure 4). In addition, carbamazepine caused the arrest of spontaneous beating when administered at a dose over 30 μM, more frequently in AM than in CT (Table 2). Carbamazepine has been reported to cause a decrease in Vmax and shortening of APD_50_ and APD_90_ in ventricular myocytes of guinea pigs at a concentration of 75 μM (Delaunois et al., 2015). Consistent with the previous study, the effects of carbamazepine on membrane potentials may be attributed to the inhibition of depolarization of ventricular myocytes through blocking of the Nav1.5 current. On the other hand, the membrane potentials of AM showed a decrease in the rising slope only at the maximal concentration. The results may be ascribed to the fact that Ca^2+^ channels, but not Na^+^ channels, contribute to membrane potentials mainly in nodal cells, and thus AM were less sensitive to CBZ. The inhibition of the Na^+^ current in both atrial-and node-type cells might cause the arrest of spontaneous beating at the maximal concentration.

### Phenytoin (Anti-epileptic Drug)

Phenytoin is an antiepileptic drug with neural Na^+^ channel blocking action, similar to the action exhibited by carbamazepine. In the present study, phenytoin showed an increase in the falling slope, a slight shortening of membrane potential duration, and arrest of spontaneous beating at concentrations higher than 30 μM in CT, while the drug caused arrests at concentrations higher than 10 μM in AM (Figure 6, supplementary Figure 4, Table 2). These results obtained with phenytoin were similar to those obtained with verapamil and diltiazem. Previous studies reported that phenytoin exhibits an inhibitory action on the cardiac Nav1.5 current and the Cav1.2 current with IC_50_ values of 72.4/120.6 μM and 21.9 μM, respectively Kramer et al., (2013), Harmer et al., (2011), demonstrating that the drug has stronger action on Cav1.2 than Nav1.5. Based on these data, it is conceivable that the results observed with the use of phenytoin in AM may be caused by its heterogenic cellular population including nodal type cells as well as atrial cells.

In the present study, various drugs demonstrated the arrest of spontaneous beatings in AM before the onset of the dysrhythmias in CT, and these results can be explained based on their actions on cardiac ion channels. These results strongly suggest that the detection of drug-induced bradyarrhythmias can be one of the important parameters in addition to the induction of tachyarrhythmias and the velocity/pattern of conduction.

## Conclusion

**FIGURE 7:**
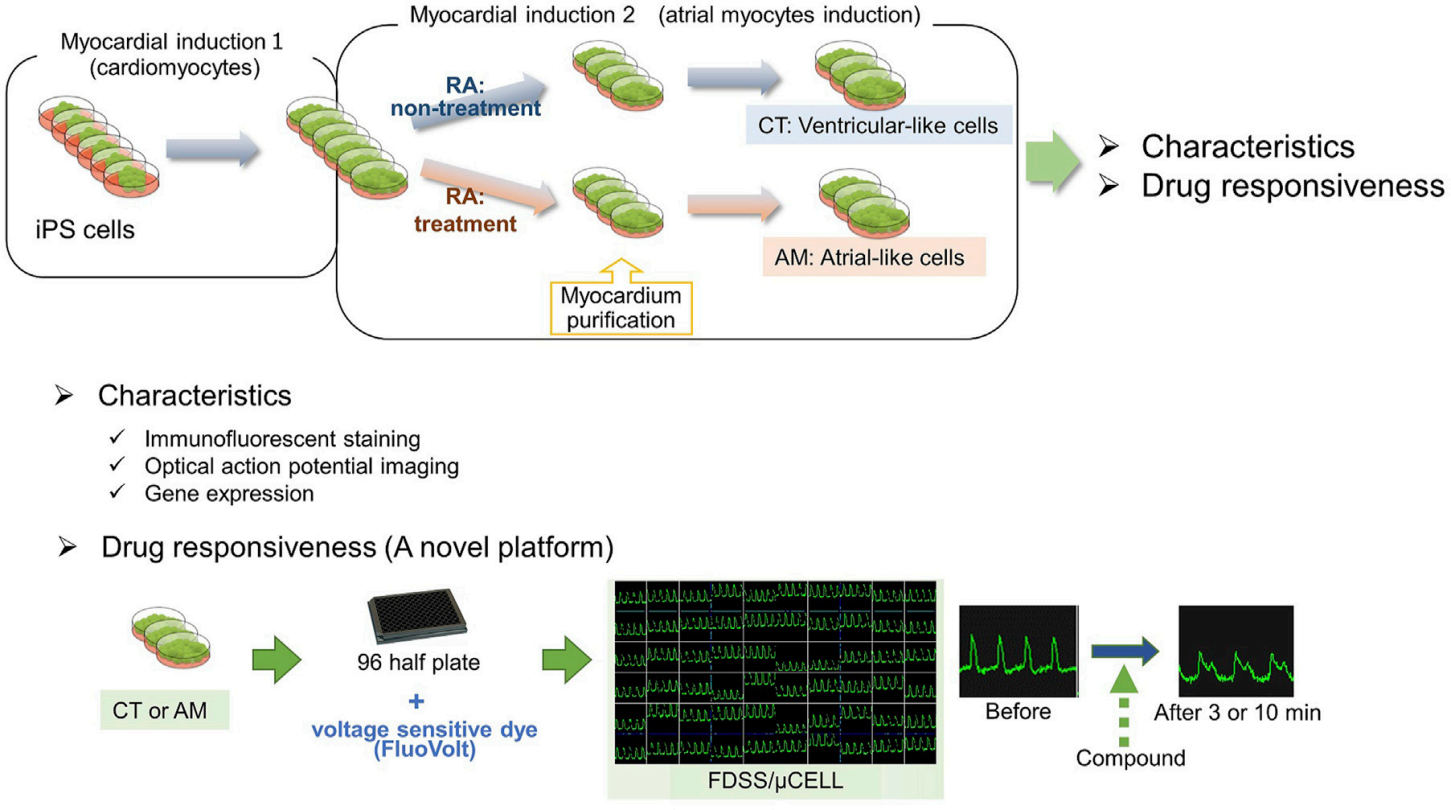
Graphic abstract. In the present study, we generated a hiPS-derived atrial-like phenotype by supplemented RA during the cardiac mesoderm induction. Thereafter, we examined the acute response to several drug administration in the hiPS-CMs with (atrial-like subtype) or without (ventricular-like subtype) treatment of RA by using a high-throughput drug evaluation system, which might be a valuable platform to detect potential risks for drug-induced atrial arrhythmias.

We successfully established a platform for human iPS-derived cardiomyocytes with atrial and nodal properties by treatment with retinoic acid (Figure 7). Membrane potential-based drug testing on the present platforms would be useful to detect propensities for drug-induced tachyarrhythmias by comparing ventricular and atrial drug responses. In addition, atrial platforms are more sensitive to bradyarrhythmias. This may be achieved with additional parameters for cardiac conduction.

These results suggest that the present evaluation system is useful for developing anti-atrial-arrhythmic drugs as well as for detecting potential risk for drug-induced atrial arrhythmias or rhythm/conduction disturbances.

## Materials and Methods

The study protocol was approved the Institutional Review Board of Osaka University and all experiments were performed under the guidelines of the Osaka University Gene Modification Experiments Safety Committee.

### Cardiac Differentiation of hiPSC-CMs

hiPSCs from a healthy human (RIKEN BRC Cell Bank, Tsukuba, Japan) were subjected to cardiac differentiation using the PSC Cardiomyocyte Differentiation Kit (ThermoFisher SCIENTIFIC). Briefly, undifferentiated hiPSCs were maintained on iMatrix-511 (Nippi, 892,021) in StemFit® AK02N medium (Ajinomoto, AK02N) and passaged enzymatically at 80%–90% confluence.

The hiPSCs were enzymatically dispersed and maintained until 80% confluence with StemFit® AK02N containing 10 µM Y-27632 (Wako, 036-24023). The next day, the culture medium was changed to StemFit® AK02N without Y-27632, which was maintained until the next passage. hiPSCs were seeded on a dish coated with Geltrex LDEV-Free Reduced Growth Factor (ThermoFisher SCIENTIFIC) at 1% concentration in PBS. When the seeded cells reached 80% confluency (day-1), the culture medium was changed to StemFit® AK02N containing Matrigel® (Growth Factor Reduced, Corning, 354,230) at 1% concentration for 24 h. On day0, the culture medium was changed to Medium A (PSC Cardiomyocyte Differentiation Kit, ThermoFisher SCIENTIFIC) containing Matrigel for 48 h. On day2, the culture medium was changed to Medium B (PSC Cardiomyocyte Differentiation Kit, ThermoFisher SCIENTIFIC) for 48 h. On day4, the culture medium was changed to cardiomyocyte maintenance medium (PSC Cardiomyocyte Differentiation Kit, ThermoFisher SCIENTIFIC) supplemented with or without 0.7 μM RA (Sigma Aldrich, R2625) for 72 h. On day 7, the culture medium was changed to cardiomyocyte maintenance medium, and change the medium every 2 days. The cells typically started spontaneous beating around 10 days of initiation of the differentiation protocol. Purification of cardiomyocytes was performed by using a non-glucose medium supplemented with 4 mM lactic acid for 4days/time, performed 2 times (from day 14 to day 18, and from day 21 to day 25), between the 2 times of purifying application, the medium was changed to cardiomyocyte maintenance medium for 72 h (from day 18 to day 21). After purification, the culture was continued in the cardiomyocyte maintenance medium until the functional analysis.

### Immunofluorescent Staining

hiPSC-derived cardiomyocytes were fixed with 4% PFA and permeabilized with 0.1% Triton-X in PBS (-) for 15 min at 4°C. Then, the cells were blocked with 5% BSA in PBS (-) for 60 min at room temperature. Primary antibodies were reacted for 24 h at 4°C, and secondary antibodies were reacted for 1 h at room temperature. Nuclei were labeled with Hoechst 33342 (Dojindo, H342). Primary antibodies were anti-Troponin T (clone 13-11) (1:200, Thermo Scientific, MA5-12960), anti-MLC2a (1:200, Synaptic Systems, 311 011), and anti-MLC2v (1:200, ProteinTech, 10906-1-AP). Secondary antibodies were Alexa Fluor 488, donkey anti-mouse IgG (HCL), Alexa Fluor 568, donkey anti-rabbit IgG (HCL), Alexa Fluor 647, donkey anti-mouse IgG (HCL). Fluorescence images were obtained using Operetta high content imaging system (PerkinElmer, Japan) and analyzed using Harmony analysis software (PerkinElmer, Japan).

### Optical Action Potential Imaging

hiPSC-CMs were loaded with a voltage-sensitive dye (FluoVolt™ Membrane Potential Kit, F10488, ThermoFisher SCIENTIFIC) for 30 min at RT. The excitation and emission wavelengths were 522 and 535 nm, respectively. Then, 10 µM blebbistatin (Wako, 027-17043), an excitation-contraction uncoupler, was applied to avert motion artifacts. All experiments were performed at 37°C under aerial conditions. Optical action potential (OAP) imaging was acquired at a sampling rate of 5 or 10 ms per frame using the MiCAM02 imaging system (Brainvision, Tokyo, Japan) equipped with a high-speed CMOS camera, alongside field-of-view and spatial resolution, which were 5.76 × 4.8 mm and 30 × 30 μm, respectively. OAP parameters including average CL, d (−F)/dt_max_, and APD, were calculated using OriginPro 8.6J software (LightStone, Tokyo, Japan).

### High-Throughput Recording of Membrane Potential Signal Recording

FluoVolt™ Membrane Potential Kit (ThermoFisher SCIENTIFIC, F10488, Massachusetts, United States) was used to measure membrane potentials. The basic procedure was performed in accordance with the manufacturer’s instructions. Briefly, the loading dye solution was adjusted with an experimental medium (1% GlutaMAX supplement (ThermoFisher SCIENTIFIC), 1% HEPES (Sigma-Aldrich, Missouri, United States), and 0.001% Pluronic F-127 (Thermo Fisher Scientific) in FluoroBrite DMEM™ (ThermoFisher SCIENTIFIC). After washing the cells twice with the experimental medium, the loading dye solution was added and loaded for 30 min at 37°C. Thereafter, the cells were washed twice with the measurement buffer (1% GlutaMAX supplement, 1% HEPES, 2% fetal bovine serum in FluoroBrite DMEM™). Fluorescence signals representing the membrane potential were measured using FDSS/μCell imaging platform (Hamamatsu Photonics K.K., Hamamatsu, Japan). MP were recorded at excitation and emission stages at wavelengths of 470 and 540 nm, respectively. Measurements were taken during the pre-test, and 10min (except for Bay K 8644, the drug response time was 3 min) post-compound addition. Stock solutions of the test compounds were prepared in 100% DMSO, and serially diluted 1/40 into compound plates for testing (0.5% DMSO was the maximum concentration in all wells). The compounds were automatically pipetted from the compound plate, and 12 μL was loaded in the wells already containing the cells with 48 μL of media. Recording data were analyzed using Waveform Analysis of Cardiomyocyte Software (Ver.1.2.1J, Hamamatsu Photonics K.K.). Data values for a well were averaged from waveforms that arose during 30 s. The beat rate (BR; beats per minute), waveform amplitude (AMP), and duration at 30, 50, and 80% of decay (PWD30, PWD 50, and PWD 80, respectively) were used as evaluation parameters. PWDs were corrected using the Fridericia formula. PWD30-80 was calculated as the difference between PWD80 and PWD30. Each parameter was calculated as percentage change (%) from the value before test compound addition.

### Data Analysis

All data are expressed as mean ± standard deviation (SD). The data were confirmed as normal distribution by the Shapiro-Wilk test. Two independent groups were compared using the Student’s t-test for homogeneity variance, and the heteroscedasticity variance by using Welch *t*-test. Multiple groups variance was compared using one-way analysis of variance (ANOVA), followed by Tukey’s test. *p* values of <0.05 were considered statistically significant. Statistical analysis was performed using JMP Pro 14.0 (SAS, Tokyo).

## Data Availability

The original contributions presented in the study are included in the article/Supplementary Material, further inquiries can be directed to the corresponding author.
