# Supplementary material for: High-Throughput Drug Screening System Based on Human Induced Pluripotent Stem Cell-Derived Atrial Myocytes ∼ A Novel Platform to Detect Cardiac Toxicity for Atrial Arrhythmias
Source: Front Pharmacol. 2021 Aug 3;12:680618. doi: 10.3389/fphar.2021.680618 (PMC8369502; doi:10.3389/fphar.2021.680618)
Supplement: Supplementary file 1 [file DataSheet1.PDF]

Supplementary Fig. 1 (related to Fig. 1)

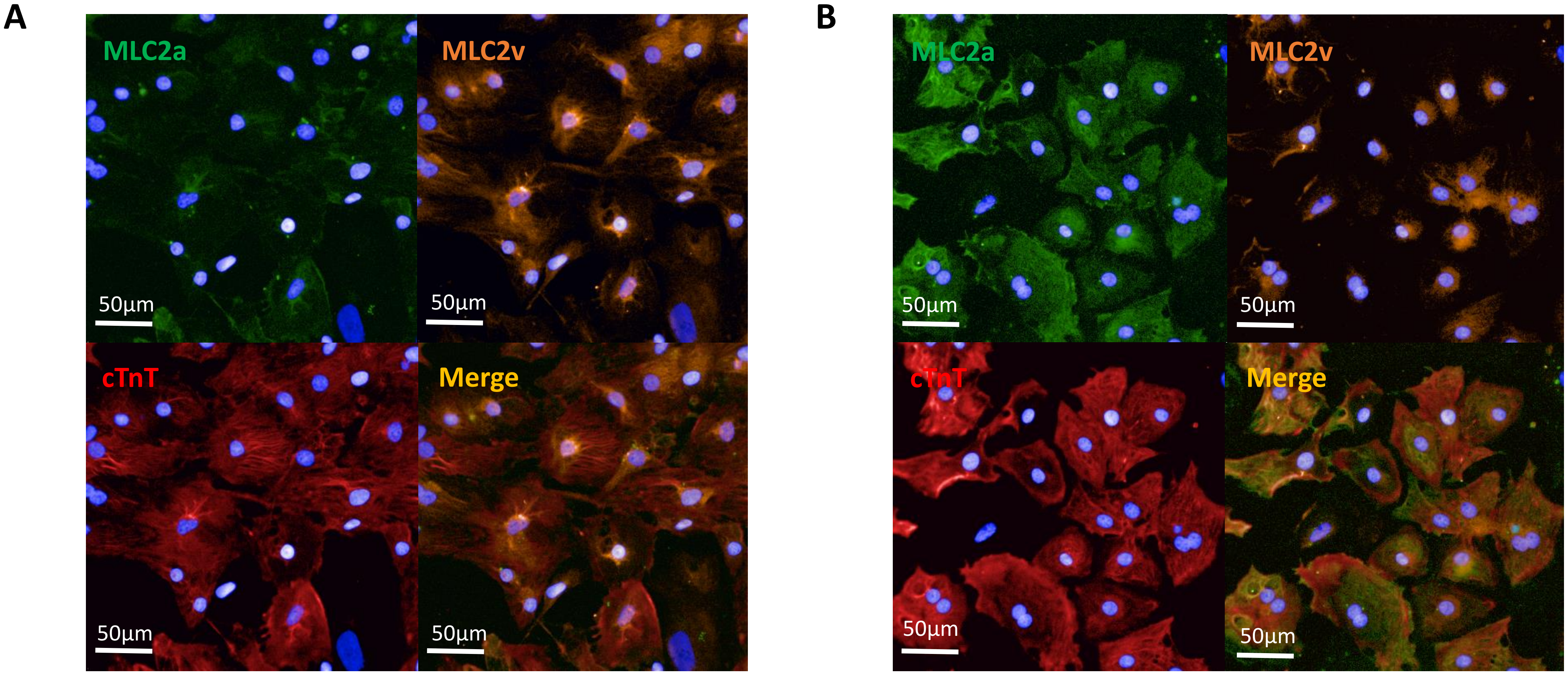

Supplementary Figure 1. Representative images for immunofluorescence analysis. Images obtained by high content imaging system. hiPS-CMs without(A,CT) or with(B,AM) RA treatment immunostained with cardiac troponin T(cTnT, red),MLC2a(green), MLC2v(orange), nuclei were stained with Hoechst 33342(blue). Scale bar, 50um.

Supplementary Fig. 2 (related to Fig. 3)

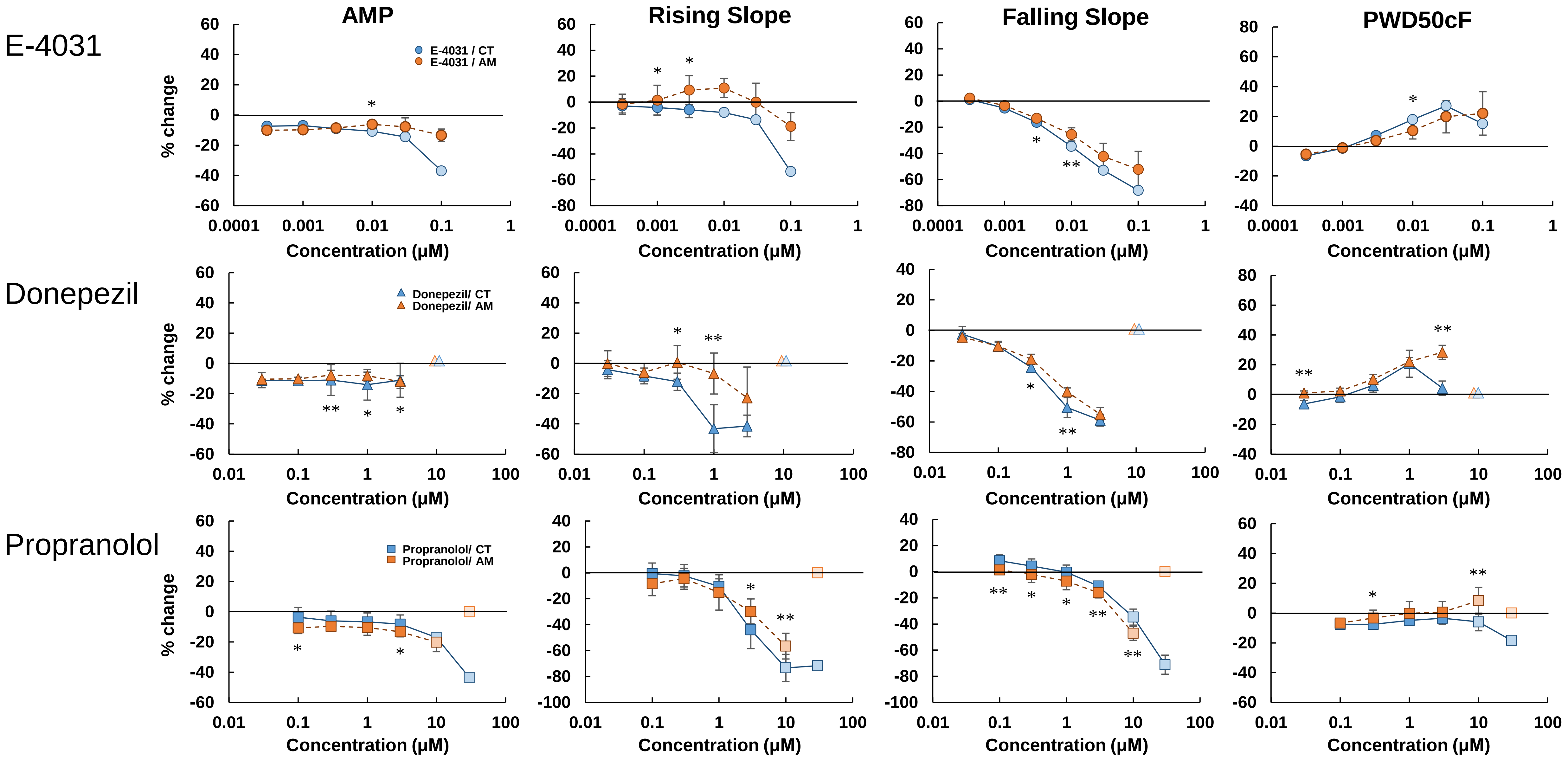

Supplementary Figure 2. Dose-response of IKr blockers in CT and AM. CT and AM were treated with various concentrations of E-4031, donepezil, and propranolol, and membrane potential (MP) was analyzed by FDSS/µCell imaging platform. Y-axis represents the percentage change from the value before test compound addition. Comparison of MP parameters: AMP, rising slope, falling slope, PWD50cF in CT and AM. Error bars represent SD of the mean from the values of independent experiments. n = 3-12. *P<0.05, **P<0.01 for comparison of drug treatment of CT vs. AM.

Supplementary Fig. 3 (related to Fig. 5)

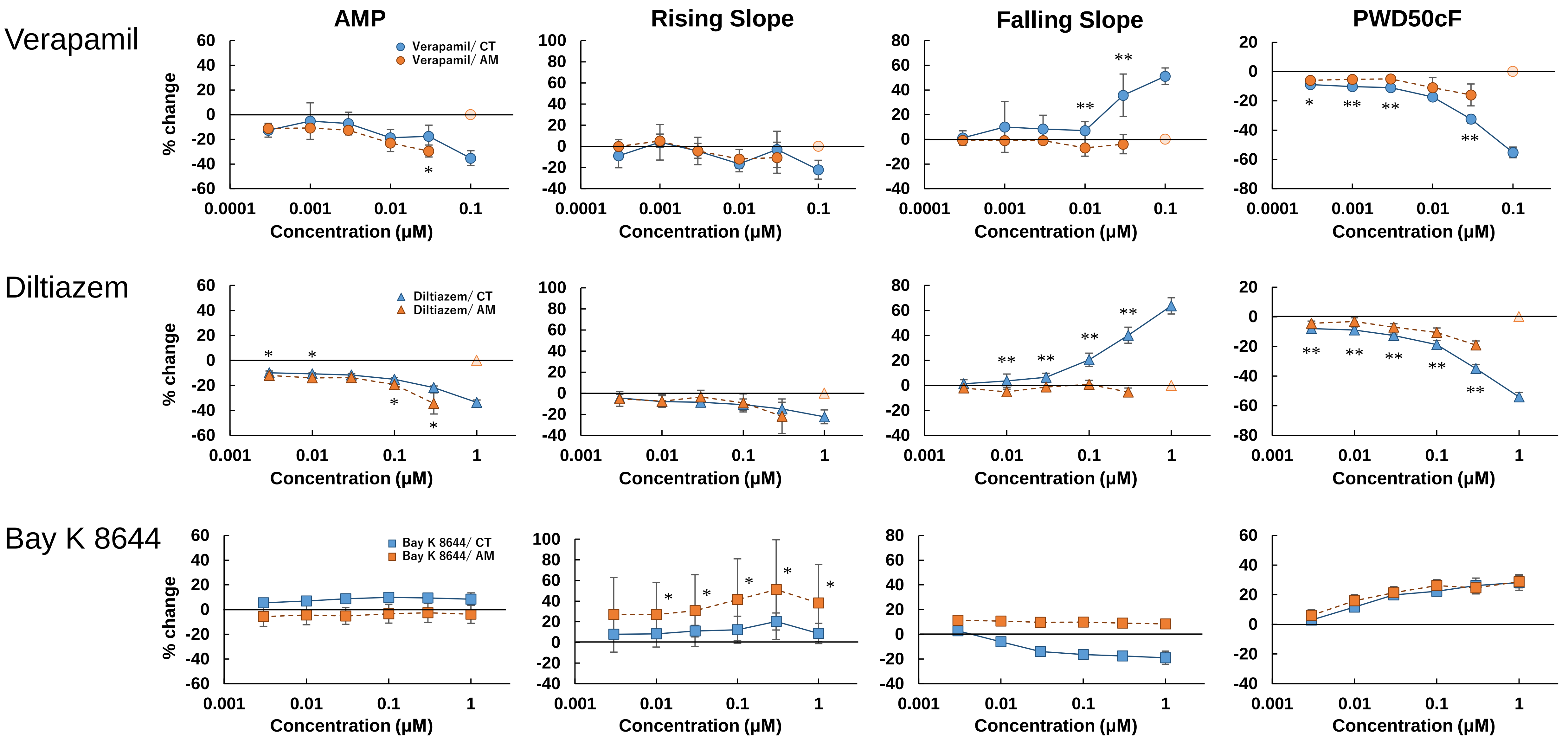

Supplementary Figure 3. Dose-response of calcium ion channel regulator in CT and AM. Comparison of MP parameters: AMP, rising slope, falling slope, PWD50cF in CT and AM after application of calcium ion channel blocker verapamil and diltiazem, as well as the selective calcium channel activator Bay K 8644. Error bars represent SD of the mean from the values of independent experiments. n = 6. *P<0.05, **P<0.01 for comparison of drug treatment of CT vs. AM.

Supplementary Fig. 4 (related to Fig. 6)

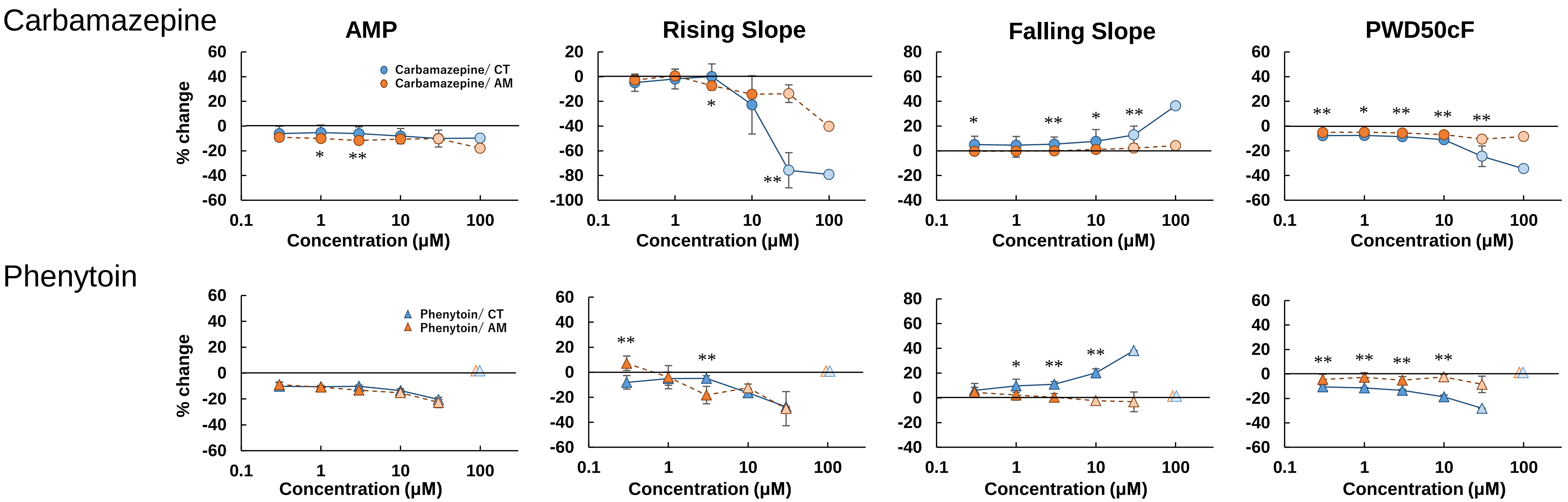

Supplementary Figure 4. Dose-response of sodium ion channel regulator in CT and AM. Comparison of MP parameters: AMP, rising slope, falling slope, PWD50cF in CT and AM after application of carbamazepine and phenytoin. Error bars represent SD of the mean from the values of independent experiments. n = 6-12. *P<0.05, **P<0.01 for comparison of drug treatment of CT vs. AM.

Supplementary Table 1 Baseline-values of membrane potential of CT and AM

Parameters	CT	AM
Number of tests	16	14
Beat rate (beat per min)	63.6 ± 11.3	102.5 ± 17.7
Amplitude	816.7 ± 230.3	557.1 ± 187.8
PWD30 (msec)	221.7 ± 29.5	105.7 ± 30.3
PWD50 (msec)	305.8 ± 36.5	164.5 ± 39.7
PWD80 (msec)	406.6 ± 36.3	265.5 ± 47.6
PWD30-40/PWD70-80	1.30 ± 0.18	0.75 ± 0.11
